# Delta-3-Carene Presented Anti-Inflammatory and Antinociceptive Properties by Modulating Leukocyte Activation in the Experimental Inflammatory Response In Vitro and In Vivo

**DOI:** 10.3390/molecules31111917

**Published:** 2026-06-02

**Authors:** Paloma Kênia de Moraes Berenguel Lossavaro, Mila Marluce Lima Fernandes, Iluska Senna Bonfá, Joyce dos Santos Lencina, Dalila dos Santos Lencina, Gabriel Silvino de Oliveira Venâncio, Fernanda Sordi Diniz, Lucas Luiz Machado, Josyelen Lousada Felipe, Luiz Alexandre Marques Wiirzler, Cândida Aparecida Leite Kassuya, Carlos Alexandre Carollo, Mônica Cristina Toffoli-Kadri, Saulo Euclides Silva-Filho

**Affiliations:** 1 Pharmaceutical Sciences, Food and Nutrition College, Federal University of Mato Grosso do Sul (UFMS), Campo Grande 79070-900, MS, Brazil; paloma.lossavaro01@gmail.com (P.K.d.M.B.L.); milamarlucefernandes@gmail.com (M.M.L.F.); iluska.senna@ufms.br (I.S.B.); joycedslencina@gmail.com (J.d.S.L.); dalila.lencina11@gmail.com (D.d.S.L.); gabriel.venancio@ufms.br (G.S.d.O.V.); fernanda.sordi@ufms.br (F.S.D.); lucaslmachado11@gmail.com (L.L.M.); josyfelipe@hotmail.com (J.L.F.); carlos.carollo@ufms.br (C.A.C.); monica.kadri@ufms.br (M.C.T.-K.); 2 Department of Pharmacology and Therapeutics, State University of Maringá (Universidade Estadual de Maringá—UEM), Maringá 87020-900, PR, Brazil; luizalexandrew@gmail.com; 3 Health Sciences College, Federal University of Grande Dourados (UFGD), Dourados 79804-970, MS, Brazil; candida2005@gmail.com

**Keywords:** delta-3-carene, terpene, inflammation, pain, leukocyte activation

## Abstract

Delta-3-carene (CAR), a monoterpene derived from plant essential oils, exhibits promising biological properties, including anti-inflammatory, antioxidative, anxiolytic, and antimicrobial activities. Therefore, this study aimed to investigate the anti-inflammatory effects of CAR by analyzing the activity of this terpene on leukocyte activation through the evaluation of cell migration in in vitro and in vivo models. Cell viability analysis demonstrated that CAR (3, 10, 30, and 90 μg/mL) exerted no cytotoxic effects and significantly reduced in vitro neutrophil chemotaxis toward *N*-formylmethionyl-leucyl-phenylalanine (fMLP). Furthermore, CAR decreased phagocytosis in zymosan-stimulated neutrophils in vitro. In Swiss mice, oral CAR treatment, at doses of 25, 50, and 100 mg/kg, reduced inflammatory and antinociceptive parameters in zymosan-induced peritonitis, carrageenan-induced paw edema and mechanical hyperalgesia, and nociception induced by acetic acid and formalin models. In the persistent inflammation model (for 21 days) induced by complete Freund’s adjuvant (CFA), daily CAR treatment (50 mg/kg) reduced paw edema and mechanical hyperalgesia in all evaluated times at 6, 11, 16, and 21 days after CFA-induced inflammation. In conclusion, our data demonstrated that CAR modifies acute and chronic inflammatory responses, highlighting its potential therapeutic application in managing inflammation and pain.

## 1. Introduction

**Figure 1:**
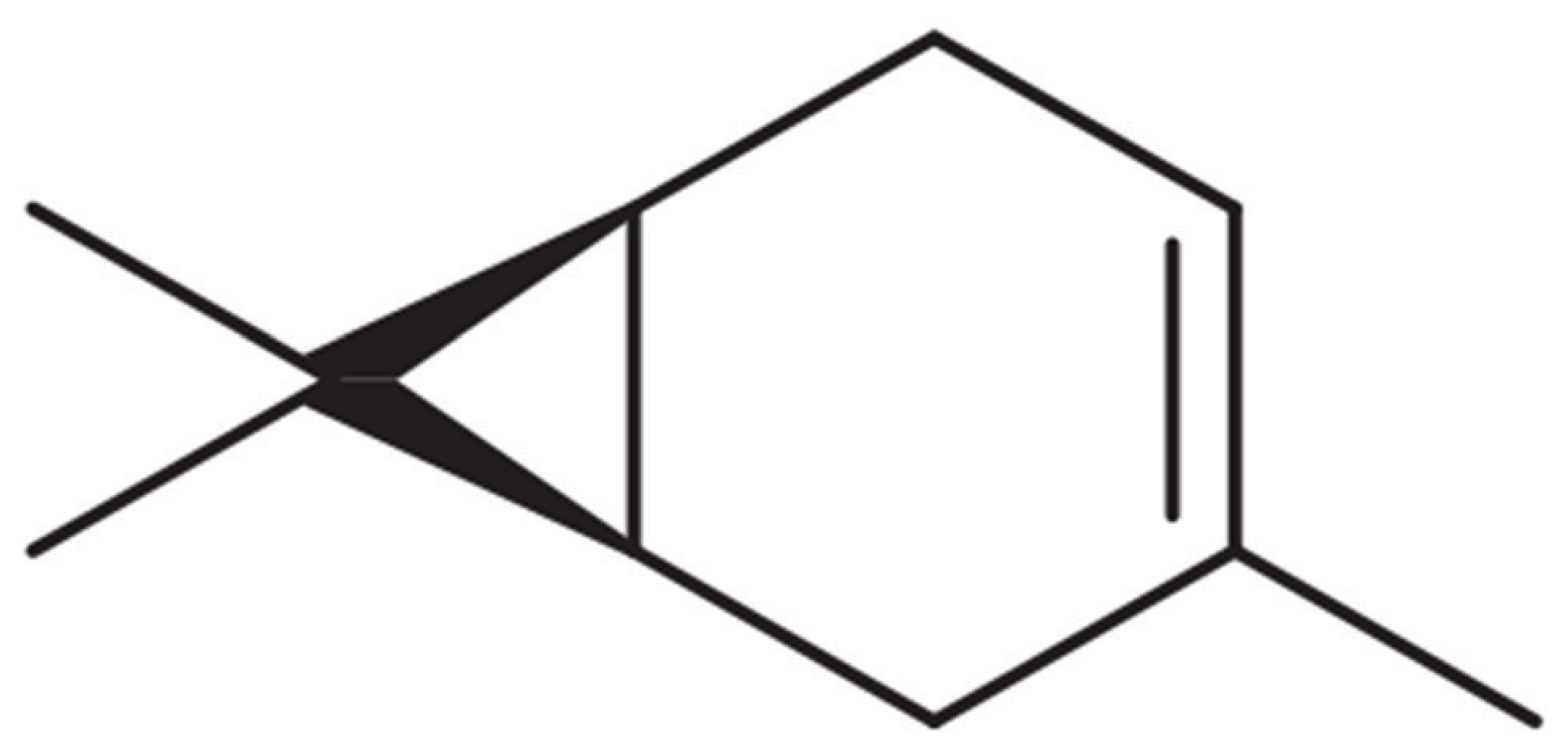
Chemical structure of delta-3-carene [8].

Delta-3-carene (CAR) (Figure 1), a bicyclic monoterpene, is widely distributed in nature, being a major component of pine (*Pinus* spp.) and spruce (*Picea abies*) oleoresin in temperate regions, as well as peppers (*Piper* spp.) in tropical and subtropical areas [1,2,3]. It is also present in various foods such as cashew (*Anacardium occidentale*), coriander seeds (*Coriandrum sativum*), ginger (*Zingiber officinale*), rosemary (*Rosmarinus officinalis*), and other spices [4]. This natural compound has attracted growing interest due to its multiple described pharmacological properties, including anti-inflammatory [5,6,7,8], anti-asthmatic [9], antimicrobial [10,11], anticancer [12], antifungal [13], sedative [14], anti-leishmania [3], and antioxidant [15] activities.

Inflammation is an essential biological response aimed at protecting the body against injuries caused by biological, chemical, or physical stimuli, promoting tissue repair and homeostasis restoration [16]. Classic signs include heat, redness, swelling, and pain, with potential loss of function in severe cases [17]. This process involves complex cellular and vascular events and can be classified as acute or chronic. In the acute phase of the inflammatory process, there is a predominance of polymorphonuclear cells (PMNs), such as neutrophils, which migrate to the injury site in response to mediators like cytokines and chemokines. When inflammation persists, evolving into the chronic phase, the cellular infiltrate changes significantly, becoming primarily composed of mononuclear cells (MNs), such as lymphocytes and macrophages. This phase is characterized by continuous tissue destruction, remodeling, and fibrosis [18,19,20].

Despite the efficacy of conventional treatments for acute and chronic inflammation, including corticosteroids, non-steroidal anti-inflammatory drugs (NSAIDs), immunosuppressants, and biological agents [21,22,23], these drugs often present significant adverse effects, especially with long-term use, such as gastric lesions [24], as well as cardiovascular and renal complications [25,26,27,28]. Given these limitations, there is increasing demand for safer, more effective, and accessible therapies.

In this context, natural compounds, such as CAR, emerge as promising alternatives. Natural products currently represent about 25% of available medicines [29,30], with monoterpenes being particularly targeted in numerous investigations due to their therapeutic versatility [31,32,33]. Thus, this study aims to investigate the anti-inflammatory effects of CAR, contributing to the development of safer and more effective therapeutic strategies for managing inflammatory processes.

## 2. Results

### 2.1. Cell Viability Analysis (MTT Assay)

In the toxicity assay, cells were exposed to different concentrations of CAR. At concentrations of 3, 10, 30, and 90 µg/mL, CAR did not decrease cell viability after 48 h of treatment. Cell viability was 100% and equivalent to the control group.

### 2.2. CAR Treatment Reduces In Vitro Neutrophil Chemotaxis

**Figure 2:**
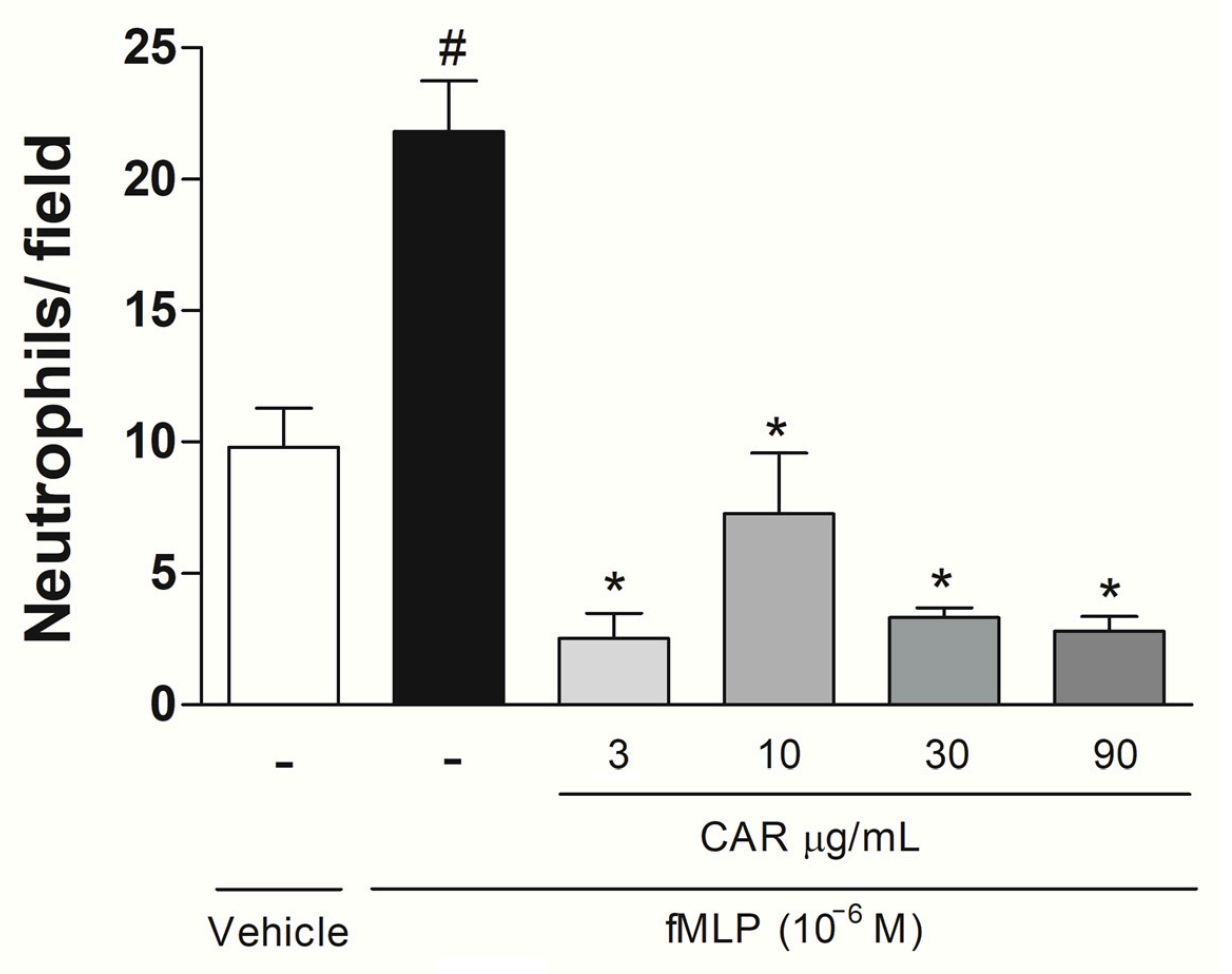
Effect of CAR on in vitro leukocyte chemotaxis. Leukocytes were obtained from zymosan-induced peritonitis (1 mg/cavity) and stimulated with fMLP (10^−6^ M) 30 min after CAR treatments at doses of 3, 10, 30, and 90 μg/mL. Results expressed in number of leukocytes/fields. Values were expressed as the mean ± S.E.M. and were representative of three independent experiments. Statistical significance: # *p* < 0.05 when compared to medium, * *p* < 0.05 when compared to the group of leukocytes stimulated with fMLP (ANOVA, Newman–Keuls test).

To investigate the direct effect of CAR on leukocyte chemotaxis, different concentrations of CAR were tested (3, 10, 30 and 90 µg/mL) in the in vitro chemotaxis assay. The fMLP induced significant leukocyte migration (20.28 ± 1.9) compared to the medium (11.66 ± 1.7). The data showed that neutrophils exposed to CAR treatment at concentrations of 3, 10, 30, and 90 µg/mL significantly reduced neutrophil phagocytosis toward fMLP (10^−6^ M) by 72.4, 53.6, 83.7, and 87.2%, respectively, compared to control group (Figure 2).

### 2.3. CAR Reduces In Vitro Phagocytic Activity of Neutrophils

**Figure 3:**
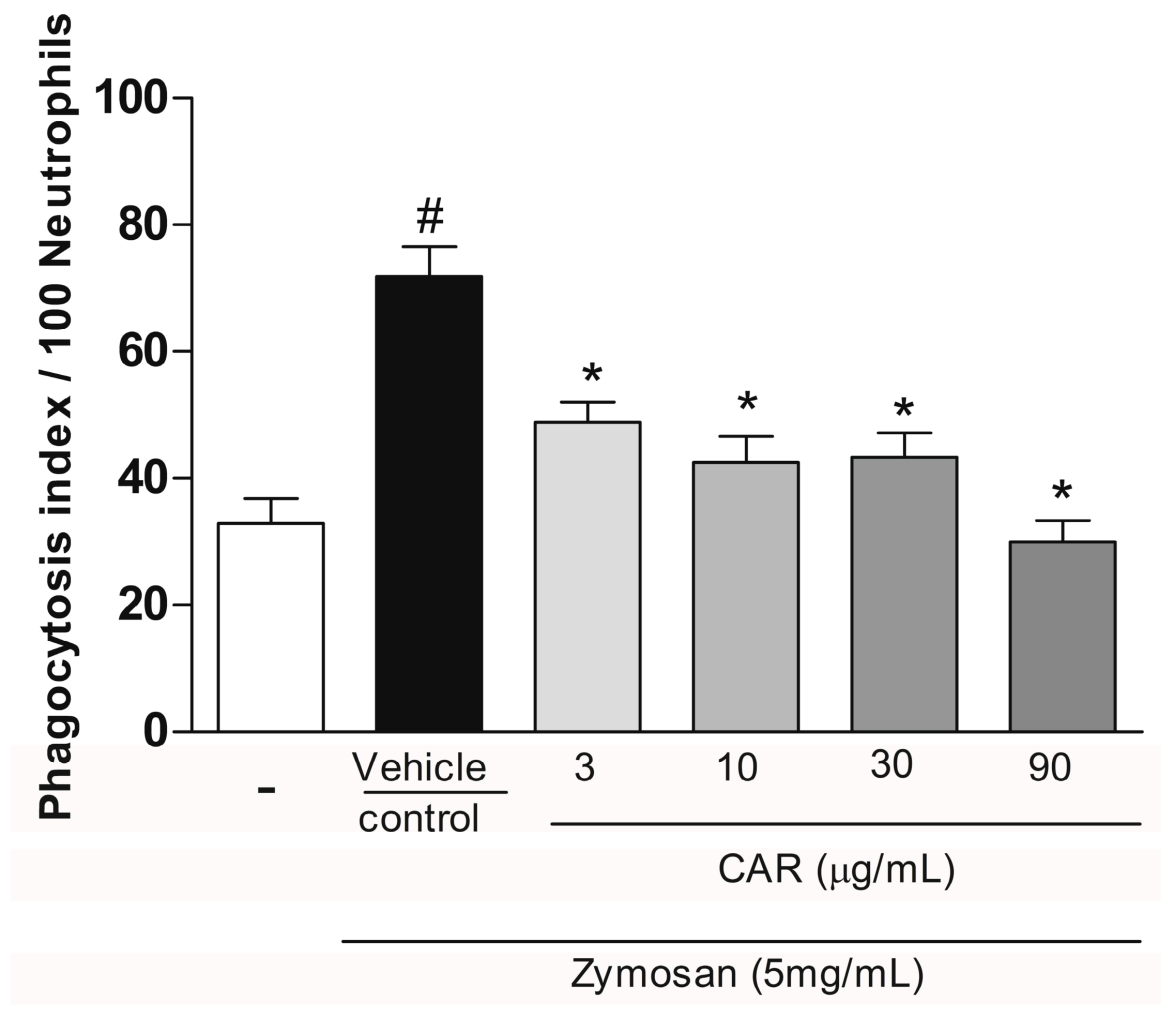
Effect of CAR on the phagocytic activity of neutrophils. Neutrophils were treated with different concentrations of CAR (3, 10, 30, and 90 µg/mL). The results are representative of three independent experiments. # *p* < 0.05 compared to medium, * *p* < 0.05 compared to the control group (ANOVA, Newman–Keuls test).

The zymosan suspension increased neutrophil phagocytosis by 71.89 ± 4.61. The results indicated that neutrophils treated with CAR at concentrations of 3, 10, 30, and 90 µg/mL exhibited a significant reduction in the phagocytosis of zymosan particles, with decreases of 68% (48.89 ± 3.13), 59.20% (42.56 ± 4.11), 57.85% (43.33 ± 3.86), and 41.67% (29.96 ± 3.34), respectively, compared to control group (Figure 3).

### 2.4. CAR Reduces Leukocyte Recruitment in the Zymosan-Induced Peritonitis Model

**Figure 4:**
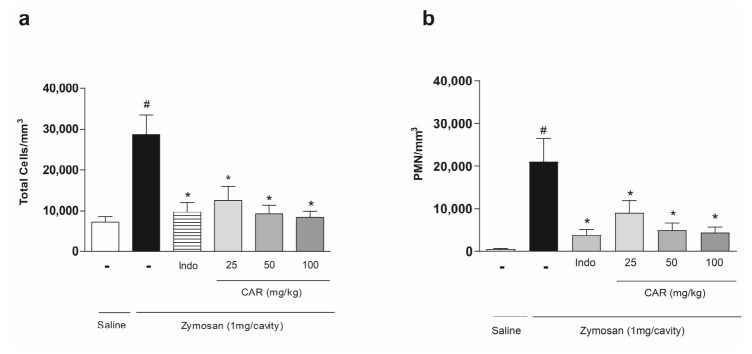
The effect of CAR treatment on migrated leukocyte number in the peritoneal cavity of Swiss mice 6 h after the zymosan injection (1 mg/cavity/i.p.). Values were expressed as the mean ± SEM (*n* = 6 animals/group). (**a**) The effect of CAR treatments on leukocyte counts 6 h after the zymosan injection in Swiss mice; (**b**) on PMN number. # *p* < 0.05 compared to saline (vehicle), * *p* < 0.05 compared to control group (ANOVA, Newman–Keuls test).

Six hours after the zymosan injection into mice peritoneal cavity, an increase in the leukocyte recruitment (28,575 ± 4950 cells/mm^3^) was observed compared with the saline group (7250 ± 1340 cells/mm^3^). Pretreatment with indomethacin (15 mg/kg) reduced the migration of leukocytes by 66.2% (9660 ± 2269 cells/mm^3^). CAR treatment (25, 50, and 100 mg/kg) significantly reduced the total leukocyte number by 56.6% (12,410 ± 3563 cells/mm^3^), 67.7% (9230 ± 2047 cells/mm^3^), and 70.7% (8370 ± 1574 cells/mm^3^), respectively, compared to control group (Figure 4a). In the leukocyte differential count, an increase in the influx of PMN cells (21,111 ± 5422 cells/mm^3^) was observed compared with the control group (476.3 ± 126.1 cells/mm^3^). It was observed that the pretreatment of animals with CAR at doses of 25, 50, and 100 mg/kg significantly reduced PMN cells recruitment 57.25% (9024 ± 2809 cells/mm^3^), 76.46% (4968 ± 1733 cells/mm^3^), and 78.93% (4448 ± 1242 cells/mm^3^), respectively (Figure 4b).

### 2.5. CAR Reduces Paw Edema Formation and Mechanical Hyperalgesia Induced by Carrageenan

**Figure 5:**
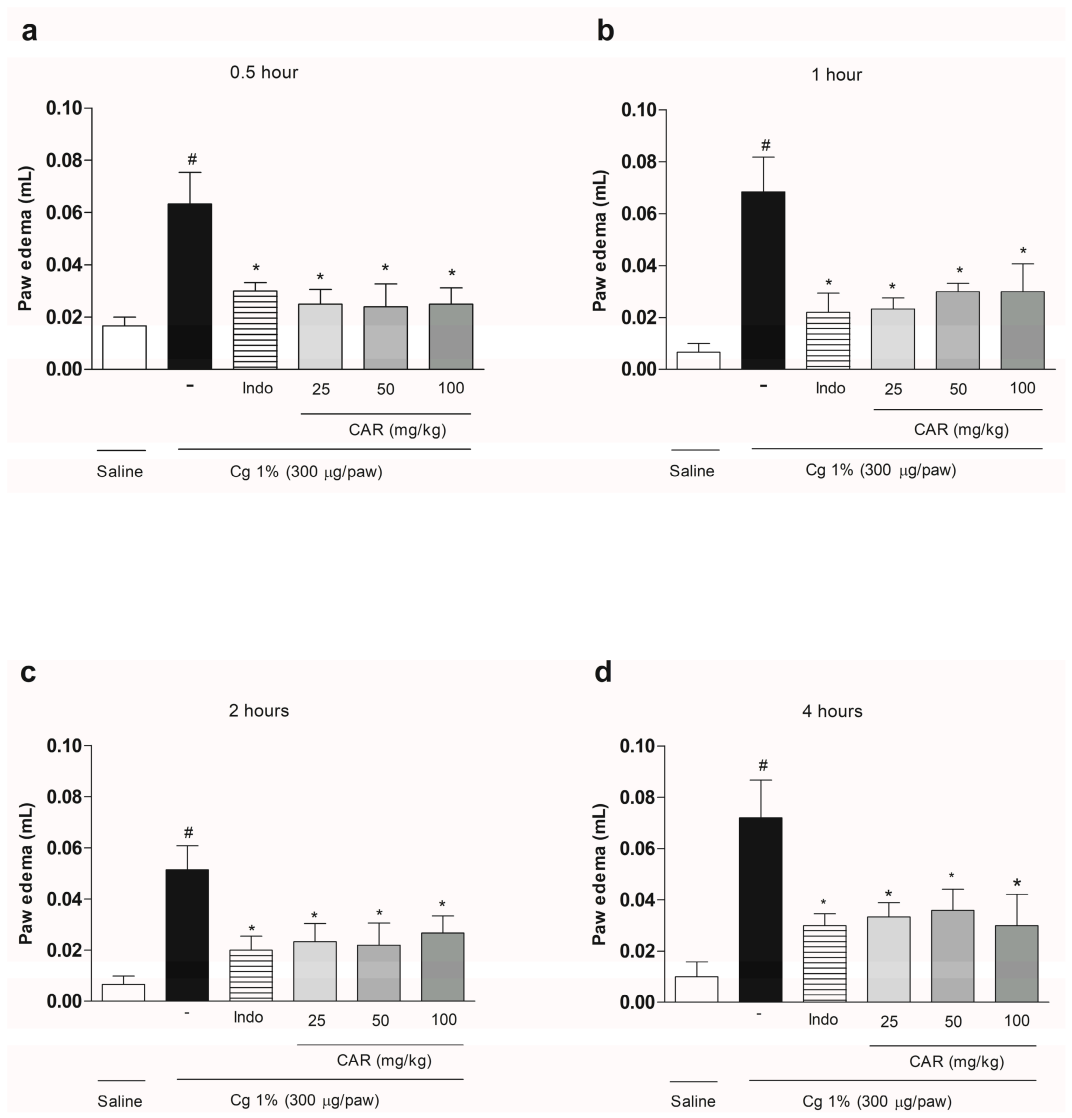
Effect of CAR treatments on carrageenan-induced paw edema in Swiss mice. Values were expressed as mean ± SEM (*n* = 6 animals/group). The figure shows the values at (**a**) 0.5 h, (**b**) 1 h, (**c**) 2 h, and (**d**) 4 h after edema induction in the control (vehicle), CAR (25, 50, and 100 mg/kg), and indomethacin (15 mg/kg) groups. # *p* < 0.05 compared to saline group, * *p* < 0.05 compared to the control group (ANOVA, Newman–Keuls test).

Cg injection caused paw edema in mice at 30 min (0.06 ± 0.01 mL; Figure 5a), 1 h (0.07 ± 0.01 mL; Figure 5b), 2 h (0.05 ± 0.01 mL; Figure 5c), and 4 h (0.07 ± 0.01 mL; Figure 5d) after stimulus compared to the saline group (0.01 ± 0.00). CAR (25, 50, and 100 mg/kg, p.o.) and indomethacin (15 mg/kg, reference drug, p.o.) treatments significantly reduced paw edema observed at 30 min, 1 h, 2 h, and 4 h (Figure 5). After Cg injection, indomethacin inhibited paw edema by 50% (0.03 ± 0.00 mL; Figure 5a), 71.4% (0.02 ± 0.00 mL; Figure 5b), 60% (0.02 ± 0.00 mL; Figure 5c), and 57.1% (0.03 ± 0.00 mL; Figure 5d). Meanwhile, pretreatment with CAR (25, 50, and 100 mg/kg) inhibited paw edema at all tested times: in 30 min, 66.6% (0.02 ± 0.00 mL; Figure 5a), 66.6% (0.02 ± 0.00 mL; Figure 5a), and 66.6% (0.02 ± 0.00 mL; Figure 5a); in 1 h, 66.66% (0.02 ± 0.00 mL; Figure 5b), 50% (0.03 ± 0.00 mL; Figure 5b), and 50% (0.03 ± 0.01 mL; Figure 5b); in 2 h, 60% (0.02 ± 0.01 mL; Figure 5c), 60% (0.02 ± 0.01 mL; Figure 5c), and 40% (0.03 ± 0.01 mL; Figure 5c); and in 4 h, 57.14% (0.03 ± 0.01 mL; Figure 5d), 42.85% (0.04 ± 0.01 mL; Figure 5d), and 57.14% (0.03 ± 0.00 mL; Figure 5d).

**Figure 6:**
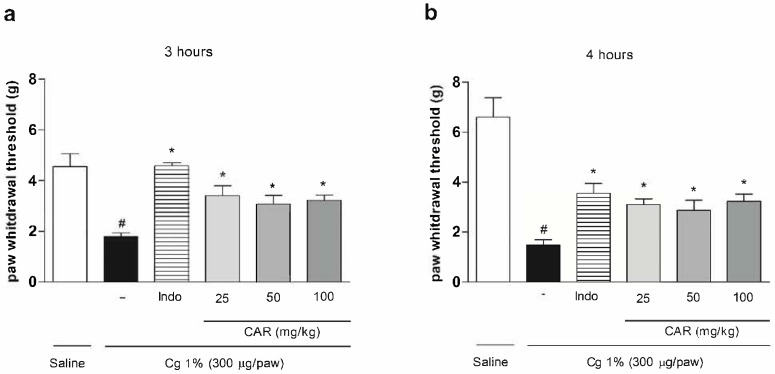
Effect of oral administration of CAR on carrageenan-induced hyperalgesia in Swiss mice. Mice were pre-treated 60 min before stimulus, p.o., with CAR (25, 50, and 100 mg/kg). The control group received water or indomethacin. Mechanical hyperalgesia was evaluated (**a**) at 3 h and (**b**) at 4 h after Cg injection (i.p.) in the paw of mice (*n* = 6 animals/group). Statistical significance: # *p* < 0.05 when compared with the saline group and * *p* < 0.05 when compared to the group of animals stimulated with Cg (ANOVA, Newman–Keuls test).

Animals in the control group showed increased mechanical hyperalgesia by 60.4% (1.80 ± 0.14 g; Figure 6a) at 3 h and 77.6% (1.48 ± 0.21 g; Figure 6b) at 4 h after stimulus, compared to the saline group (4.55 ± 0.51 g and 6.59 ± 0.78 g). After 3 h and 4 h Cg injection, indomethacin (15 mg/kg) reduced mechanical hyperalgesia by 39.21% (4.59 ± 0.12 g; Figure 6a) at 3 h and 41.57% (3.56 ± 0.37 g; Figure 6b) at 4 h. The treatment with CAR (25, 50, and 100 mg/kg) reduced mechanical hyperalgesia by 52.94% (3.40 ± 0.39 g; Figure 6a), 58.44% (3.08 ± 0.33 g; Figure 6a), and 55,90% (3.22 ± 0.21 g; Figure 6a) at 3 h and by 47.89% (3.09 ± 0.24 g; Figure 6b), 51.56% (2.87 ± 0.39 g; Figure 6b), and 45.82% (3.23 ± 0.29 g; Figure 6b) at 4 h, respectively. Both were compared to the water-treated control group. There was no statistically significant difference between the doses (*p* > 0.05) and between the indomethacin-treated at the same periods.

### 2.6. CAR Reduces Abdominal Contortion Induced by Acetic Acid

**Figure 7:**
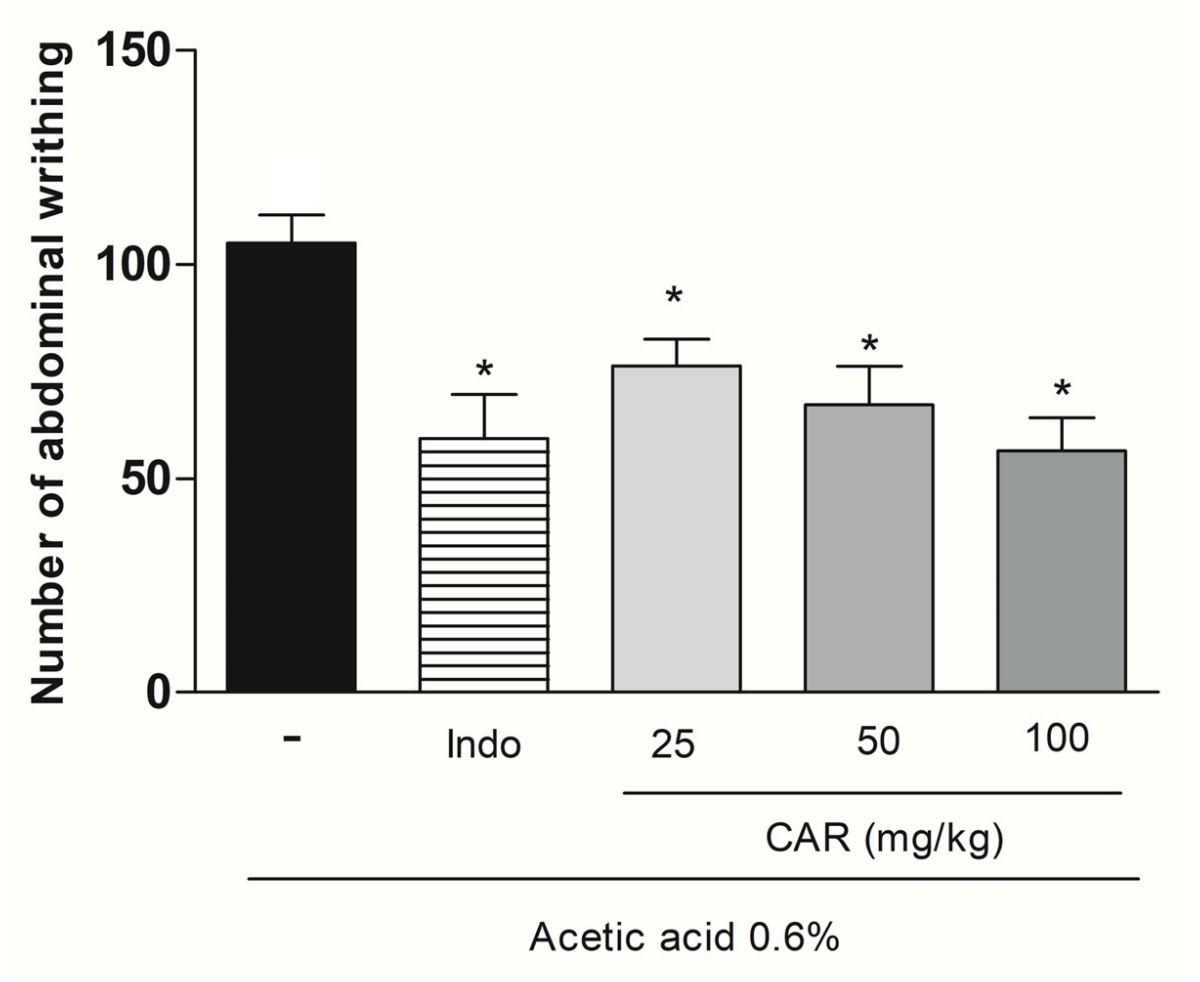
Effect of CAR treatment on acetic acid-induced abdominal writhing. Animals were pre-treated 60 min before challenge, p.o., with CAR (25, 50, and 100 mg/kg), while animals in the control group received water or indomethacin; mice (*n* = 6 animals/group), stimulus—injection via i.p. 0.6% acetic acid (10 mL/kg), and the number writhing recorded during 30 min. Results were expressed as mean ± SEM. * *p* < 0.05 compared to the control group (ANOVA, Newman–Keuls test).

The acetic acid injection promoted an increase in abdominal writhing (105.0 ± 6.56) in animals that received the vehicle. The indomethacin (reference group, 15 mg/kg) treatment promoted a significant reduction of 43.49% (59.33 ± 10.19) in writhing compared to the control group. The treatment with CAR (25, 50, and 100 mg/kg) reduced the number of abdominal writhing by 27.30% (76.33 ± 6.29), 36% (67.17 ± 9.0), and 46.28% (56.40 ± 7.73), respectively, compared to vehicle (Figure 7). These results showed a significant antinociceptive effect regarding all CAR doses administered.

### 2.7. Formalin-Induced Pain or CAR on the Formalin-Induced Paw Licking Response in Mice

**Figure 8:**
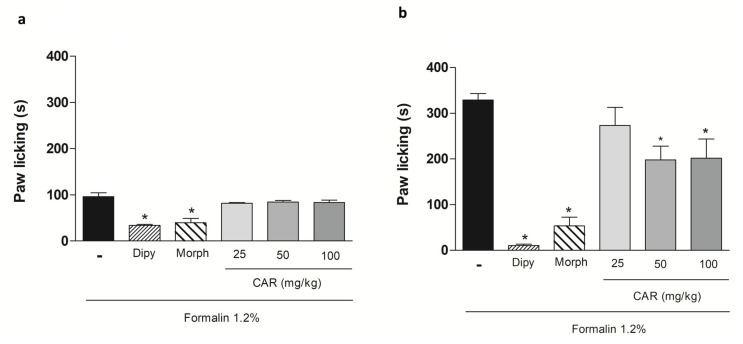
Formalin-induced paw licking after treatment with CAR. Animals were tested in the first 5 min (**a**) first phase and 15–30 min (**b**) second phase of nociception in mice. Animals were pre-treated (p.o.) with saline (0.9% NaCl), dipyrone, or morphine 30 min before and CAR 60 min before intraplantar injection of formalin (40 µL). Columns represent the mean ± SEM (*n* = 6 animals/group) of licking time in seconds. Results were expressed as mean ± SEM. * *p* < 0.05 compared to the control group (ANOVA, Newman–Keuls test).

The paw licking time in phase I was 96.19 ± 8.30 s (Figure 8a) and in phase II 328.9 ± 14.17 s (Figure 8b) in control group. Oral administration of CAR (25, 50, and 100 mg/kg) reduce paw licking only in the second phase at doses of 50 and 100 mg/kg. In phase II, CAR treatment reduced the paw licking time by 32.92% (220.6 ± 10.0 s) and 46.18% (177.0 ± 33.2 s), compared to control group (Figure 8a). The morphine group showed a reduction in the paw licking time of the animals by 58.93% (39.50 ± 9.54 s) in phase I and 83.79% (53.31 ± 19.04 s) in phase II. The dipyrone group exhibited inhibition of the paw licking time in both phases by 64.96% (33.7 ± 2.3 s) and 96.95% (10.0 ± 3.5 s).

### 2.8. CAR Treatment Reduces Paw Edema and Mechanical Hyperalgesia in the CFA-Induced Persistent Inflammation Model

**Figure 9:**
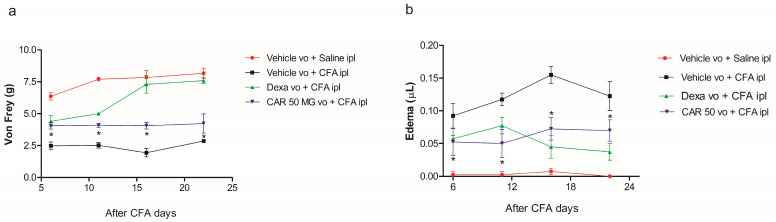
Effects of chronic oral administration of CAR (50 mg/kg) on CFA-induced (**a**) mechanical hyperalgesia and (**b**) edema formation at 6, 11, 16 and 21 days after CFA injection. The animals received a daily oral administration of CAR (50 mg/kg) once a day for 21 days in the CFA model. Values are expressed as the mean ± SEM (*n* = 6 animals/group). * *p* < 0.05 compared to the control group (two-way ANOVA, Newman–Keuls test).

The treatment with CAR (50 mg/kg) reduced edema formation and mechanical hyperalgesia in CFA-induced persistent inflammation. The effects of CAR were observed on mechanical hyperalgesia and paw edema over 21 days at time points of 6, 11, 16, and 21 days in this model. On day 16, we observed the highest peak of hyperalgesia (1.94 ± 0.33) (Figure 9a) and edema formation (0.15 ± 0.00) (Figure 9b), compared to the naive group. CAR treatment at a dose of 50 mg/kg reduced mechanical hyperalgesia on days 6, 11, 16 (97.91%), and 21. As expected, dexamethasone reduced mechanical hyperalgesia at all evaluated time points (Figure 9a). CAR treatments reduced edema formation on days 6, 11, 16 (53.33%), and 21. Dexamethasone reduced edema formation at all evaluated time points (Figure 9b).

## 3. Discussion

The monoterpene delta-3-carene (CAR) has demonstrated remarkable therapeutic potential due to its important biological activities reported in the recent literature, including anti-inflammatory and antinociceptive effects [8,34,35,36,37,38,39]. Although recent studies have characterized the anti-inflammatory and antinociceptive activities of CAR, its potential to specifically modulate leukocyte recruitment remains poorly explored [34,36,39]. Our study aimed to systematically investigate CAR ability to interfere with the mechanisms of leukocyte migration by combining in vitro and in vivo approaches.

The manifestations of inflammatory response include vasodilation, increased vascular permeability, and leukocyte migration to the affected area [40]. Neutrophil migration to the inflammatory site is a multifactorial event involving several steps, such as neutrophil rolling and adhesion to endothelial cells, followed by chemotaxis induced by a group of inflammatory mediators known as chemoattractants [41]. Leukocyte chemotaxis is an initial process in the inflammatory response, triggered by the release of signaling molecules at the site of injury, such as the fMLP peptide produced by bacteria that cause inflammation, or substances released by resident cells; for example, components of the complement system (C5a) and lipid mediators, such as leukotriene B4 (LTB4), which can stimulate neutrophils and monocytes [41]. However, the excessive activation and dysregulated migration of these leukocytes can lead to the release of reactive oxygen species and proteolytic enzymes, amplifying the inflammatory response and causing collateral tissue damage, such as chronic inflammation [42]. Inhibition of leukocyte recruitment can reduce sequelae associated with inflammatory conditions [43]. Therefore, substances capable of interfering with the chemotactic process may hold promise for the treatment of inflammatory diseases.

Our data demonstrated that the incubation of neutrophils with CAR (3, 10, 30, and 60 µg/mL) significantly reduced the in vitro chemotaxis of these cells stimulated by fMLP. The fMLP induces leukocyte chemotaxis by binding to a G protein-coupled receptor, activating pathways such as Mitogen-Activated Protein Kinase (MAPK) and phosphoinositide 3-kinase (PI3K), which stimulate the production of pro-inflammatory cytokines, such as interleukin-1β (IL-1β), interleukin-8 (IL-8), and Tumor Necrosis Factor (TNF) [44]. Studies have shown that monoterpenes modulate this in vitro chemotaxis by regulating the release of these cytokines [41,44,45,46,47].

Neutrophil recruitment mediated by chemotaxis is followed by the phagocytic capacity of these cells against the aggressor agent in the inflammatory focus. Phagocytosis is essential for the microbicidal activity of neutrophils [48,49]. Thus, neutrophil phagocytosis was evaluated in vitro using zymosan. All concentrations of CAR significantly reduced neutrophil phagocytosis. This mechanism may be associated with the blockade of multiple signaling pathways, including MAPKs and NF-κB, which are involved in the anti-inflammatory effect of monoterpenes [31,44,50,51]. The reduction in neutrophil chemotaxis and phagocytic activity observed with CAR does not result from cytotoxic effects, as demonstrated by the maintenance of cell viability in the MTT assay.

Based on the in vitro chemotaxis results, we evaluated the anti-inflammatory effect of CAR in a zymosan-induced peritonitis model. Experimental peritonitis induced by zymosan (a β-glucan-rich component derived from the cell wall of Saccharomyces cerevisiae) in mice occurs through an inflammatory response triggered by activation of the toll-like receptor 2 (TLR2) pathway in leukocytes, such as macrophages and neutrophils [51]. Once bound to the receptor, there is significant recruitment of leukocytes into the peritoneal cavity, activation of NF-κB, and subsequent production of pro-inflammatory mediators, including IL-1β, IL-6, TNF, chemokines, and nitric oxide (NO) [15,52,53]. Therefore, this model has been used to investigate mechanisms of acute inflammation [54]. During the inflammatory response, neutrophils are the first cells to migrate to the affected tissue, playing a crucial role in the initial cellular recruitment [40]. Our results demonstrate that CAR (at doses of 25, 50, and 100 mg/kg, p.o.) reduced leukocyte migration into the peritoneal cavity. These findings are consistent with the literature, which shows that CAR, in a murine asthma model, suppressed inflammatory cytokines and pulmonary epithelial thickness, which may influence cell migration [9]. Furthermore, CAR affects the migration of LPS-activated RAW264.7 cells, which may be related to its anti-inflammatory activity [7]. As zymosan-induced peritonitis is widely associated with the TLR2/NF-κB pathway [51] and considering the previously reported ability of CAR to modulate cytokine release [9], the observed reduction in leukocyte migration could be involved with these signaling cascades. However, further studies are needed to confirm this mechanism.

In an experimental model of acute inflammation, the effects of CAR on paw edema and mechanical hyperalgesia induced by Cg were evaluated in mice. A single oral administration of CAR (25, 50, and 100 mg/kg) significantly inhibited both paw edema and mechanical hyperalgesia at all time intervals analyzed. These findings are consistent with previous studies. Huang et al. (2019) [7] reported that essential oils from Gynura procumbens inhibited xylene-induced ear edema, and isolated CAR reduced formalin-induced edema and increased the pain threshold in the hot plate test. Similarly, oils from Bupleurum gibraltaricum demonstrated anti-inflammatory activity in a Cg-induced edema model [5], and a similar pattern was observed by Zhang et al. (2025) [8], in which CAR showed significant anti-inflammatory activity by reducing Cg-induced edema in animal models and suppressing cyclooxygenase-2 (COX-2) expression in LPS-stimulated RAW264.7 cells.

The Cg-induced inflammation model is biphasic. The initial phase can be observed between 30 and 60 min after injection and involves the intense release of vasoactive amines, such as histamine, serotonin, bradykinin, and kinins. In this phase, the increased in vascular permeability and blood flow mediated by vasodilation occur mainly due to the release of serotonin and histamine, leading to the formation of local edema [53,55]. The late phase, between 2 and 4 h, is mainly mediated by eicosanoids (leukotrienes and prostaglandins), NO, and COX-2 [3,56,57,58]. This model promotes primary sensitization of sensory neurons, leading to hyperalgesia, which is an increased response to painful stimuli. Prostaglandin E2 (PGE2) acts as the main mediator of painful hypersensitivity, both in peripheral and central mechanisms. This mediator induces primary hyperalgesia at the injury site by enhancing responses to subthreshold stimuli and lowering the activation threshold of nociceptive neurons [53]. Our results, together with others in the literature, indicate that the anti-edematogenic and antinociceptive effects of CAR may involve the attenuation of the production/release of vasoactive amines and PGE2 through the inhibition of COX-2 [7,8,31,59,60,61].

CAR demonstrated significant antinociceptive and anti-inflammatory effects in the experimental models evaluated. To better characterize these properties, complementary nociception tests were conducted, including the acetic acid-induced abdominal writhing model and the formalin test. The acetic acid-induced abdominal writhing test is a highly sensitive and non-selective experimental model for evaluating anti-inflammatory substances. This test involves the activation of visceral somatic receptors and local inflammatory processes mediated by PGs, bradykinins, and inflammatory cytokines, such as TNF, IL-6, IL-1β, and IL-8 [62], which sensitize nociceptive fibers. The CAR treatment (25, 50, and 100 mg/kg, p.o.) significantly reduced the number of abdominal writhing induced by acetic acid, indicating its potential analgesic effect, which may act through the reduction in pro-inflammatory cytokines as previously described for other monoterpenes using the same test, such as bornyl acetate [18], estragole [63,64], linalool, and essential oils containing linalool [65,66]. Furthermore, Basholli-Salihu et al. (2017) [6] demonstrated that essential oils from *Pinus mugo* containing CAR as their main constituent reduced IL-6 secretion.

The formalin test is a well-established model for investigating antinociceptive activity, allowing the distinction between the acute neurogenic phase (0–5 min) and the late inflammatory phase (15–30 min) [67]. The initial phase reflects the direct activation of nociceptive fibers and peripheral neural transmission, with substance P and bradykinin mediating pain activation. In contrast, the late phase is characterized by the release of inflammatory mediators, such as bradykinin, histamine, and prostaglandins. Morphine, an opioid analgesic, and dipyrone, an antipyretic and analgesic, were effective in both phases of the test, reducing the nociceptive behavior induced by formalin. CAR treatment, at doses of 50 and 100 mg/kg, promoted antinociceptive effects exclusively during the second phase of the formalin test, a pattern consistent with other monoterpenes, such as piperitenone oxide [68] and miternol [69,70], suggesting a predominantly anti-inflammatory mechanism that inhibits the synthesis or release of arachidonic acid and its metabolites, including prostaglandins and leukotrienes [62]. The absence of action in the neurogenic phase indicates that CAR does not act through direct blockade of acute nociceptive transmission, thus distinguishing it from analgesics such as opioids.

Neutrophils play a crucial role in the pathogenesis of chronic inflammatory and autoimmune diseases through tissue infiltration, contributing to conditions such as rheumatoid arthritis [66]. In the process of chronic inflammation, a characteristic transition is observed in the inflammatory cell population, with the progressive replacement of PMNs (predominant in the acute phase) by mononuclear cells, including macrophages, lymphocytes, plasma cells, and fibroblasts [41]. Based on these results, we evaluated the effects of CAR in persistent inflammation induced by CFA (complete Freund’s adjuvant). Intraplantar injection of CFA promotes the development of edema and increased tissue volume, accompanied by thermal and mechanical hypersensitivity [41,71]. These changes occur due to the accumulation of inflammatory cells and the consequent increase in the expression of pro-inflammatory cytokines (IL-1β, IL-6, and TNF) in paw tissues, which induce prolonged neuronal depolarization and, consequently, greater pain sensitivity [71,72]. The results showed a reduction in edema at all evaluated time points in animals treated with CAR (50 mg/kg) compared to the control group. The same was observed in mechanical hyperalgesia. This response pattern suggests that the effects of CAR are predominantly related to the modulation of inflammatory mediators.

The results obtained with CAR in the CFA model revealed a pharmacological action profile distinct from other monoterpenes [73,74,75,76], characterized by a gradual reduction in edema and mechanical hyperalgesia. This temporal pattern suggests that CAR modulates not only the prostaglandin cascade [9,67] but also other mechanisms involved in the chronification of the inflammatory process, such as potential effects on tissue remodeling. A study demonstrated that low concentrations of CAR stimulate osteoblast differentiation [77], indicating that its mechanism of action may involve the suppression of pro-inflammatory cytokines and COX-2 expression, as well as the activation of regenerative pathways. This would be consistent with our findings of a late phase reduction in CFA-induced inflammation, a stage in which repair processes are activated.

This pharmacological distinction is particularly relevant given the therapeutic limitations of anti-inflammatory drugs in the management of chronic pain [78,79]. The action profile of CAR, although pharmacokinetically challenging [80], points to potential advantages in the treatment of persistent inflammatory conditions [53], warranting further studies for better characterization its molecular mechanisms.

## 4. Materials and Methods

### 4.1. Chemicals and Drugs

Delta-3-carene (CAR) was purchased from Elevation Terpenes (Loma Linda, CA, USA), and analyzed by GC–MS (Figure S1 and Table S1, Supplementary Materials). The following reagents were obtained from Sigma-Aldrich (St. Louis, MO, USA): [3-(4,5-dimethylthiazol-2-yl)-2,5-diphenyl-2Htetrazolium bromide] (MTT), zymosan (Zy), N-formyl methionyl leucyl phenylalanine (fMLP), ethylenediamine tetraacetic acid (EDTA), indomethacin, dexamethasone, carrageenan (Cg), complete Freund’s adjuvant (CFA), dimethyl sulfoxide (DMSO), formalin, acetic acid, polyoxyethylene sorbitan monolaurate (Tween 20), and sterile saline (0.9% NaCl). Additionally, dipyrone (Farmace^®^, Tianguá, CE, Brazil), morphine (Cristália^®^, Itapira, SP, Brazil), xylazine, and ketamine (Syntec^®^, Santana de Parnaíba, SP, Brazil) were used.

### 4.2. Animals

The present study used male Swiss mice (*n* = 6 animals per experimental group; mean weight 20–30 g; age 8 to 9 weeks), obtained from the Animal Facility of the Federal University of Mato Grosso do Sul (UFMS), Campo Grande, MS, Brazil. The animals were kept in a room with a controlled temperature (22 ± 2 °C), relative humidity (60 ± 5%), 12/12 h light/dark cycles, and had access to standard chow (Nuvital CR) and water ad libitum. No animal was excluded from the study. Six hours before the start of each experiment, the animals received only water to avoid food–drug interactions. The experimental procedures were approved by the Animal Ethics Committee of UFMS (protocol number 1.288/2023). The animals were euthanized with an anesthetic overdose of ketamine and xylazine (300 mg/kg and 30 mg/kg, respectively, i.p.).

### 4.3. In Vitro Assays

#### 4.3.1. Leukocyte Preparation

To perform the in vitro tests, leukocytes were obtained from the peritoneal cavity of male Swiss mice 4 h after the intraperitoneal zymosan injection (1 mg/animal). To obtain the cells, the mice’s peritoneal cavities were washed with 1 mL of phosphate-buffered saline (PBS) solution containing ethylenediamine tetraacetic acid (EDTA). The collected peritoneal exudate was centrifuged (1000 rpm/10 min/4 °C), the supernatant was discarded, and the pellet was resuspended in an RPMI1640 medium containing 0.1% of bovine serum albumin (BSA). The leukocyte viability was verified by the trypan blue method, which was above 98%. Additionally, a differential cell count was performed on 95% of the leukocytes obtained corresponded to neutrophils.

#### 4.3.2. Cell Viability Analysis

Methylthiazolyldiphenyl-tetrazolium bromide (MTT) assay was performed according to Mosmann (1983) [81], adapted from de Freitas Junior et al. (2022) [44]. The MTT assay is based on the mitochondrial enzyme reduction in the tetrazolium dye to detect and determine cell viability. The neutrophils were plated at a density of 2 × 10^6^ cells/well in a volume of 100 μL RPMI medium (supplemented with 10% of fetal bovine serum (FBS) and penicillin 100 U/mL + streptomycin 100 μg/mL) into 96-well plates. After 90 min exposure to CAR (3, 10, 30, or 90 μg/mL) or vehicle (0.1% Tween 20 solution, used as control), 10 μL of MTT (5 mg/mL) stock solution was added to each well. After 2 h of incubation at 37 °C, the supernatant was removed and 200 μL of DMSO were added to each well. Cells were incubated at 25 °C for a further 10 min, and the absorbance was measured using an ELISA reader (HumaReader HS^®^, Wiesbaden, Germany) at 540 nm. The results were presented as percentage values (%) of viable cells relative to the control group.

#### 4.3.3. In Vitro Neutrophil Chemotaxis

The neutrophil chemotaxis test was conducted according to the protocol described by Silva-Filho et al. (2016) [45]. To evaluate the effects of CAR on chemotaxis, neutrophils were harvested from the peritoneal cavity. The cells were resuspended in RPMI 1640 medium supplemented with 0.1% bovine serum albumin (BSA) to achieve a final concentration of 1 × 10^6^ cells/mL. The chemotaxis assay was carried out using a 48-well microchemotaxis plate (Neuro Probe, Gaithersburg, MD, USA), with the compartments separated by a polyvinylpyrrolidone-free polycarbonate membrane (with a pore size of 5 µm). The chemoattractant, N-formyl methionyl leucyl phenylalanine (fMLP, 10^−6^ M), and the negative control (RPMI 1640 vehicle) were placed in the lower chamber. Meanwhile, a neutrophil suspension was pre-treated for 30 min with CAR (3, 10, 30, and 90 µg/mL) and then added to the upper chamber. The plate was incubated at 37 °C in 5% CO_2_ for 1 h. Following incubation, the membrane was rinsed with PBS and stained with Hema3^®^. For each well, the membrane area was examined under a light microscope (Leica AG, Heerbrugg, Switzerland) for neutrophil counts in five randomly selected fields. The data were presented as the mean number of neutrophils per field.

#### 4.3.4. In Vitro Phagocytic Activity of Neutrophils

This assay was performed as previously described by Silva-Filho et al. (2016) [45]. Neutrophils were obtained from the peritoneal cavity and plated at a density of 2 × 10^6^ cells/mL in RPMI 1640 medium. Therefore, 1 mL of this suspension of cells was exposed to CAR at concentrations of 3, 10, 30, and 90 µg/mL or the vehicle for 30 min. Zymosan particles used for induction of phagocytosis were previously opsonized by incubation with mouse plasma to 10% for 30 min. After the treatment period, cells were centrifuged (Heraeus Megafuge 16R centrifuge, Thermo Scientific, Madrid, Spain) and resuspended in 1 mL of RPMI medium containing 10% of mouse plasma and 10 μL of zymosan opsonized solution (5 mg/mL) for 30 min at 37 °C in 5% CO^2^. Then, cells were fixed and stained. Counting was performed in a specific optical (Leica AG, Heerbrugg, Switzerland) device, and the results were expressed as the number of phagocytosis neutrophils per 100 neutrophils.

### 4.4. In Vivo Assays

#### 4.4.1. Leukocyte Recruitment in Zymosan-Induced Peritonitis Model

The leukocyte influx assay was performed based on the protocol described by de Freitas Junior et al. (2022) [44] with slight modifications. Male Swiss mice (*n* = 6 animals per group) received an oral (p.o., gavage) pretreatment with different doses of CAR (25, 50, and 100 mg/kg) dissolved in saline solution (0.9% NaCl) containing 1% Tween 20. A control group was established, which received only the vehicle (0.9% NaCl with 1% Tween 20, p.o.), and a reference group treated with indomethacin (15 mg/kg, p.o., Sigma Aldrich, St. Louis, MO, USA) diluted in 0.9% NaCl. After 60 min, the inflammatory process was induced through an intraperitoneal (i.p.) injection of 0.5 mL of zymosan in all groups, except for the basal control (negative vehicle), which received 0.5 mL of 0.9% NaCl (i.p.). Six hours after stimulation, the animals were euthanized through an anesthetic overdose via i.p. injection of a combination of ketamine hydrochloride and xylazine hydrochloride, with the doses increased threefold compared to surgical anesthesia (300 mg/kg and 30 mg/kg, respectively). After euthanasia, the peritoneal cavity was washed with 1 mL of heparinized PBS solution (0.1%) containing 3% bovine serum albumin (BSA) to collect the peritoneal exudate. To determine the cell count, 10 μL of a cellular exudate aliquot was added to 190 μL of Turk’s solution, and the total count was performed in a Neubauer chamber under light microscopy (Leica AG, Heerbrugg, Switzerland). For the differential analysis, the remaining exudate was centrifuged at 1000 rpm for 5 min. The cell pellets were resuspended in 200 μL of 3% BSA, fixed on slides, and subsequently stained with Hema3^®^. The results were expressed as the number of leukocytes per cavity.

#### 4.4.2. Paw Edema and Mechanical Hyperalgesia Induced by Carrageenan

One day before the experiment, the basal mechanical threshold was determined (mechanical sensitivity) using a digital mechanical analgesimeter (Von Frey, Insight^®^, Ribeirão Preto, SP, Brazil). Male Swiss mice were orally treated with CAR (25, 50, and 100 mg/kg, p.o., gavage), indomethacin (15 mg/kg), or vehicle (0.9% NaCl solution containing 1% of tween 20—control group) (*n* = 6 animals/group) 1 h before paw edema induction with intraplantar injection of 40 μL of the 1% carrageenan (Cg) solution (300 μg/paw). Carrageenan was injected in the right hind paw of all animals, and NaCl 0.9% was injected into the contralateral paw at the same volume and considered as time zero. Edema was evaluated at 0.5, 1, 2, and 4 h, while mechanical hyperalgesia was evaluated at 3 and 4 h after Cg injection. Paw edema evaluation was performed using a plethysmometer (Insight^®^, Ribeirão Preto, SP, Brazil) [82,83]. The evaluation of mechanical hyperalgesia was performed after the animals were placed in a containment box with support for the analgesimeter test. The animals were allocated for 30 min to adapt by decreasing exploratory behavior to support four paws on the base. To measure the nociceptive mechanical sensitivity threshold (g) of the paw that received the Cg injection, a digital analgesimeter (Von Frey, Insight^®^, Ribeirão Preto, SP, Brazil) [84] was used as a pressure transducer, which records the applied force (in grams) until the moment of paw withdrawal.

#### 4.4.3. Acetic Acid-Induced Abdominal Writhing

The assay was performed according to previously described [85]. Animals were treated with CAR (25 mg/kg, 50 mg/kg, and 100 mg/kg), indomethacin (15 mg/kg), or vehicle (*n* = 6 animals/group). After 60 min of treatment, 500 μL 0.6% acetic acid was injected intraperitoneally (i.p.). The number of abdominal contortions presented by the animal was recorded 30 min after the stimulus injection. Results were expressed in the number of writhing.

#### 4.4.4. Formalin-Induced Acute Nociception in Mice

The assay was performed according to previously described [82]. Animals were orally pre-treated with vehicle (saline), dipyrone (reference drug, 50 mg/kg, p.o.), or CAR (25, 50, and 100 mg/kg, p.o.) 60 min before the injection formalin, and pre-treated with morphine (opioid antinociceptive positive control, 5 mg/kg i.p.) 30 min before challenge (*n* = 6 animals/group). The animals received an intraplantar injection (40 μL) of 1.2% formalin diluted in saline solution in the right hind paw. The naive group received an intraplantar injection (40 μL) of saline solution (0.9%). The time that the animal spent licking or biting its paws was measured between 0–5 min (neurogenic phase) and 15–25 min (inflammatory phase) after sub-plantar injection of 40 μL formalin. The results were expressed in seconds.

#### 4.4.5. Model of Persistent Inflammation Induced by Complete Freund’s Adjuvant (CFA)

The persistent edema and mechanical hyperalgesia model induced by complete Freund’s adjuvant (CFA) was used to evaluate the effect of CAR on the persistent inflammatory response. At time zero, 20 μL of CFA (suspension of killed Mycobacterium tuberculosis in oil) was injected into the mice’s right hind paw (intraplantar), and 20 μL of saline was injected into both paws. The animals were treated with CAR (50 mg/kg), dexamethasone, or vehicle (*n* = 6 animal/group) 24 h after the injection of CFA for 21 days, once a day. Mechanical hyperalgesia and paw edema were measured 6, 11, 16, and 21 days after CFA injection. The evaluation of mechanical hyperalgesia was performed using a digital analgesimeter (Von Frey, Insight^®^, Ribeirão Preto, SP, Brazil), as described earlier. Edema formation was measured with a plethysmometer (Insight^®^, Ribeirão Preto, SP, Brazil), expressed as the percentage of reduction in edema in treatment mice compared to that in the control mice [86].

### 4.5. Statistical Analysis

Data were expressed as the mean ± SEM for each experimental group. The results were statistically analyzed by using a one-way variance analysis (ANOVA), followed by the Newman–Keuls test and two-way ANOVA, and then by the Newman–Keuls post-test. The percentage of inhibition was calculated in relation to the control group. Differences were considered significant at *p* < 0.05. All statistical analyses were performed with GraphPad Prism 9.0 (GraphPad Software, San Diego, CA, USA).

## 5. Conclusions

In conclusion, CAR has demonstrated anti-inflammatory and antinociceptive effects, primarily by modulating leukocyte response in vitro and in vivo, in addition to exhibiting anti-edematogenic and anti-hyperalgesic properties. Among the doses tested, 50 mg/kg (p.o.) was identified as the optimal dose, as it provided the best balance of efficacy across all experimental models without signs of toxicity. With a safe and multifactorial pharmacological profile, CAR is a promising candidate for treating acute and chronic inflammation, as well as associated pain. However, further studies are needed to clarify its molecular targets and enable its clinical application.

## Data Availability

The data presented in this study are available on request from the corresponding author.

## Supplementary Materials

The following supporting information can be downloaded at: https://www.mdpi.com/1420-3049/31/11/1917/s1, Figure S1: GC–MS chromatogram of the delta-3-carene sample used in this study, showing delta-3-carene as the major constituent and minor peaks assigned to β-pinene and (−)-limonene, together with one unidentified compound; Table S1: GC–MS identification of compounds detected in the delta-3-carene sample based on retention indices and mass spectral data.

## Abbreviations

The following abbreviations are used in this manuscript:

CAR	Delta-3-Carene
CFA	Complete Freund’s Adjuvant
fMLP	N-Formyl-Methionyl Leucyl Phenylalanine
PBS	Phosphate-Buffered Saline
EDTA	Ethylenediamine Tetraacetic Acid
MTT	[3-(4,5-Dimethylthiazol-2-yl)-2,5-Diphenyl-2hTetrazolium Bromide]
Zy	Zymosan
Cg	Carrageenan
DMSO	Dimethyl Sulfoxide
PMS	Polymorphonuclear
MN	Mononuclear
NSAID	Non-Steroidal Anti-Inflammatory Drugs
LTBA	Leukotriene B4
COX-2	Cyclooxygenase-2
NO	Nitric Oxide
PGE2	Prostaglandin E2
MAPK	Mitogen-Activated Protein Kinase
PI3K	Phosphoinositide 3-Kinase
IL	Interleukin
TNF	Tumor Necrosis Factor
TRL2	Toll-Like Receptor 2
