# Supplementary material for: Delta-3-Carene Presented Anti-Inflammatory and Antinociceptive Properties by Modulating Leukocyte Activation in the Experimental Inflammatory Response In Vitro and In Vivo
Source: Molecules. 2026 Jun 2;31(11):1917. doi: 10.3390/molecules31111917 (PMC13439039; doi:10.3390/molecules31111917)
Supplement: Supplementary file 1 [file molecules-31-01917-s001.zip › molecules-4301434-supplementary.pdf]

Supplementary Material

Delta-3-Carene Presented Anti-Inflammatory and Antinociceptive Properties by Modulating Leukocyte Activation in the Experimental Inflammatory Response In Vitro and In Vivo

Paloma Kênia de Moraes Berenguel Lossavaro [1], Mila Marluce Lima Fernandes [1], Iluska Senna Bonfá [1], Joyce dos Santos Lencina [1], Dalila dos Santos Lencina [1], Gabriel Silvino de Oliveira Venâncio [1], Fernanda Sordi Diniz [1], Lucas Luiz Machado [1], Josyelen Lousada Felipe [1], Luiz Alexandre Marques Wiirzler [2], Cândida Aparecida Leite Kassuya [3], Carlos Alexandre Carollo [1], Mônica Cristina Toffoli-Kadri [1] and Saulo Euclides Silva-Filho [1,*]

[1] Pharmaceutical Sciences, Food and Nutrition College, Federal University of Mato Grosso do Sul (UFMS), Campo Grande 79070-900, MS, Brazil; paloma.lossavaro01@gmail.com (P.K.d.M.B.L.); milamarlucefernandes@gmail.com (M.M.L.F.); iluska.senna@ufms.br (I.S.B.); joycedslencina@gmail.com (J.d.S.L.); dalila.lencina11@gmail.com (D.d.S.L.); gabriel.venancio@ufms.br (G.S.d.O.V.); fernanda.sordi@ufms.br (F.S.D.); lucaslmachado11@gmail.com (L.L.M.); josyfelipe@hotmail.com (J.L.F.); carlos.carollo@ufms.br (C.A.C.); monica.kadri@ufms.br (M.C.T.-K.)

[2] Department of Pharmacology and Therapeutics, State University of Maringá, (UEM), Maringá 87020-900, PR, Brazil; luizalexandrew@gmail.com

[3] Health Sciences College, Federal University of Grande Dourados (UFGD), Dourados 79804-970, MS, Brazil; candida2005@gmail.com

* Correspondence: saulo.e@ufms.br

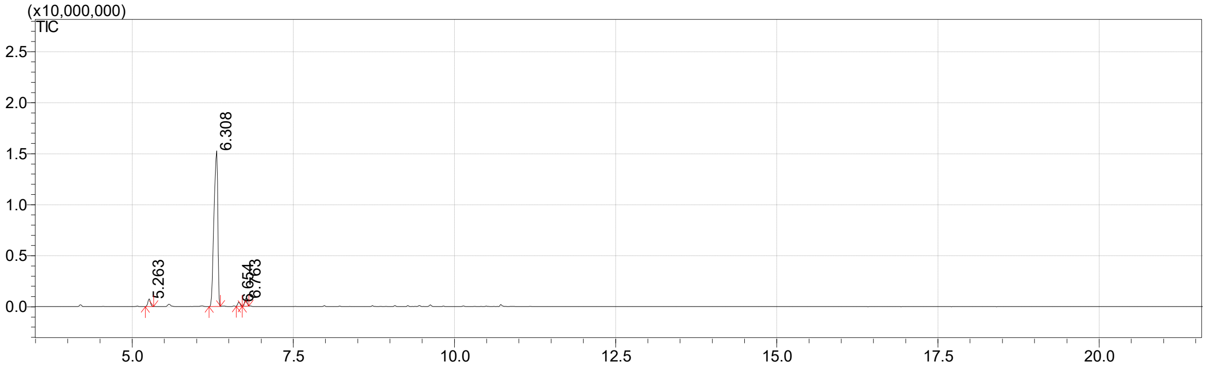

Figure S1. GC–MS chromatogram of the delta-3-carene sample used in this study, showing delta-3-carene as the major constituent and minor peaks assigned to β-pinene and (−)-limonene, together with one unidentified compound.

Table S1. GC–MS identification of compounds detected in the delta-3-carene sample based on retention indices and mass spectral data.

Peak	Retention time (min)	RI[a]	RI-lit[b]	Peak area (%)	Assigned compound
1	5.263	975	977	3.04	β-Pinene
2	6.308	1026	1026	92.51	Delta-3-Carene
3	6.654	1042	—	1.55	Unknown
4	6.763	1047	1044	2.90	(−)-Limonene

[a] Experimental retention index calculated on a non-polar Rtx®-5MS column.

[b] Literature retention index.

Experimental

GC–MS analysis was performed using a Shimadzu GCMS-QP2010 Plus system equipped with an Rtx®-5MS capillary column (Restek, 30 m × 0.25 mm i.d., 0.25 μm film thickness; Cat. No. 12623, Serial No. 893262). The injector temperature was set at 250 °C and analyses were carried out in split mode. Helium was used as the carrier gas under linear velocity control, with a column flow of 1.13 mL min^{-1} and a linear velocity of 39.0 cm s^{-1}. The oven temperature program was as follows: 75 °C for 6 min, increased at 20 °C min^{-1} to 170 °C and held for 5 min, then increased at 5 °C min^{-1} to 260 °C and held for 35 min. The MS interface and ion source temperatures were both set at 260 °C. Data were acquired in scan mode from m/z 45 to 500, with a solvent cut time of 3.0 min. To calculate retention indices, a homologous series of n-alkane standards (C9–C30, Sigma) was injected under the same chromatographic conditions.
